# Can a bigger budget shrink the bill at the bedside? Public health financing as a route to lower out-of-pocket spending in India

**DOI:** 10.3389/fpubh.2026.1903360

**Published:** 2026-07-20

**Authors:** Shyamkumar Sriram

**Affiliations:** Department of Rehabilitation and Health Services, College of Public Affairs and Health Sciences, University of North Texas, Denton, TX, United States

**Keywords:** catastrophic health expenditure, essential medicines, government health expenditure, health financing, India, out-of-pocket expenditure, strategic purchasing, universal health coverage

## Abstract

Out-of-pocket payments still finance approximately two-fifths of India’s health spending and push an estimated 55 million people into poverty each year. However, the past decade tells a more hopeful story: as the government’s share of total health expenditure rose from 28.6 to 48%, the out-of-pocket share fell from 64.2 to 39.4%. This Perspective argues that the most dependable lever India holds against household health costs is its own budget, and that the country’s own experience, rather than theory, makes the case. It sets out why public financing lowers out-of-pocket spending through free provision, pooled prepayment, and the state’s power as a price-setting purchaser, and why government health expenditure, at approximately 1.9% of gross domestic product, falls short of the 2.5% target India set for itself. The article identifies the largest and most neglected burden in outpatient care and medicines, which absorb approximately two-thirds of out-of-pocket spending yet lie outside the flagship hospitalization scheme, and points to a proven domestic remedy in Tamil Nadu’s pooled free-medicines model. Reaching 2.5% of GDP is necessary but not sufficient; how the money is spent, and how cleanly it is purchased, determines how much reaches families.

## Introduction

When an Indian family faces a serious illness, the question that often decides its future is not whether care exists, but rather, what that care will cost out of pocket. Out-of-pocket payments still account for approximately 39% of the country’s total health spending (1), and their consequences are counted in livelihoods rather than in ledgers. A nationally representative analysis of household survey data estimated that such payments push approximately 55 million Indians into poverty each year, and that approximately one in six households incurs catastrophic health expenditure (2). These are distinct measures: impoverishment counts those driven below the poverty line by medical bills, while catastrophic expenditure counts those whose health spending exceeds a certain percentage of their household consumption. A recent systematic review estimated the number crossing that catastrophic threshold at close to 90 million people (3). Financial protection, then, is not a refinement of the health system; for tens of millions, it is the line between recovery and ruin, and between staying afloat and falling into debt that outlasts the illness itself.

This Perspective presents one argument against the grain of a debate crowded with insurance products, digital platforms, and private partnerships: the most dependable instrument India holds against this burden is its own public budget. The claim does not rest on theory. It rests on what India has actually done over the past decade, on a close reading of where the remaining burden sits, and on a remedy the country has already implemented within its own borders.

## A decade of progress, and what drove it

**Figure 1:**
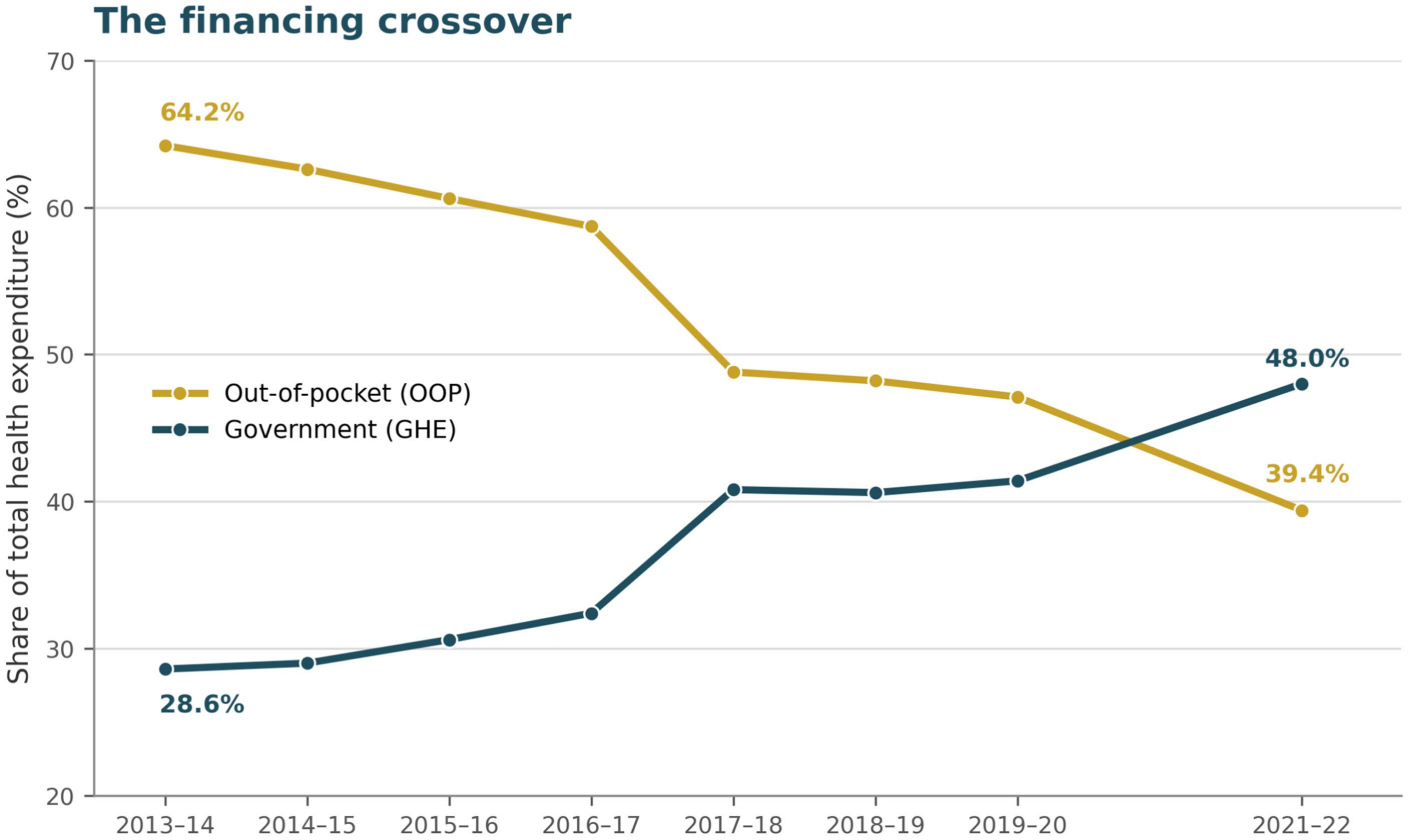
The financing crossover. Annual National Health Accounts estimates, 2013–14 to 2021–22 (2020–21 not plotted). As the government’s share of total health expenditure rose, the out-of-pocket share fell; the sharpest fall in the out-of-pocket share occurred between 2016–17 and 2017–18, before the pandemic, and the two shares cross toward the end of the period. The government and out-of-pocket shares do not sum to 100%: the balance is financed by social health insurance, prepaid private spending, external funds, and other sources. Source: National Health Accounts estimates (1).

Over the past decade, India crossed a financing threshold that had eluded it for a generation. Between 2013–14 and 2021–22, the government’s share of total health expenditure climbed from 28.6 to 48%, while the out-of-pocket share fell from 64.2 to 39.4% (1) (Figure 1). The two lines cross by the end of that period, meaning that the state now finances more of the nation’s health than households do. The ministry releasing the accounts was unusually direct about the connection, noting that the rise in government spending carries an immediate implication for the financial hardship borne by families (1). Other independent studies have found similar results, placing the out-of-pocket share at approximately 47% in 2019–20, before it fell further (3).

The pattern is hard to miss: as public money went in, the household bill came down, and over the decade, the two moved together in the direction predicted by the financing argument. It would be easy to attribute the shift to the pandemic, when emergency spending surged, but Figure 1 traces the full annual series, showing that the decline was gradual rather than occurring in a single step during the pandemic. Indeed, the sharpest fall in the out-of-pocket share came between 2016–17 and 2017–18, several years before the pandemic, which points to a structural change rather than a one-off response to a crisis (1). That distinction matters for policy: a crisis effect would fade, whereas a structural one can be built upon. Aggregate co-movement of this kind cannot by itself prove causation, but it is a strong, theory-consistent association, and India’s experience tracks the central proposition of health financing economics on its own population at scale, and with remarkably little fanfare. The task is to see the achievement clearly enough to build on it rather than mistake it for a problem already solved.

## The limits of the decade’s progress

Honesty about the gains sharpens rather than softens the argument. A 39.4% out-of-pocket share is a triumph against India’s own past, but it remains high by international standards, approximately double the level at which the majority of high-performing systems sit, and far above the point at which catastrophic spending becomes rare. Part of the recorded decline also reflects the denominator: when total spending swells during a pandemic, the out-of-pocket share can fall even if households are no better off in absolute terms. The gains are unevenly distributed, too, and tend to be thinnest where the need is greatest, in poorer states and rural districts and among the older adults, who carry the heaviest burden of chronic illness and have the least insurance (2, 3). None of this undercuts the central finding that public money lowers the household bill. It does mean the progress is fragile, reversible, and incomplete, which is why the financing argument has to be pressed, not rested upon.

## Why public money lowers the household bill

Why should public and out-of-pocket spending move so reliably in opposite directions? Three mechanisms do the majority of the work, and they compound. The first is substitution: when the state provides or subsidizes a service, the patient does not pay for it at the point of care. The second is pooling. Tax-funded and prepaid financing spreads the cost of illness across the healthy and the sick, and across a lifetime, so that no single household has to absorb the full cost of a catastrophic event in the month it happens. Risk that is unbearable for one family becomes manageable for a population, which is the entire logic of insurance, delivered here through the budget rather than the premium.

The third mechanism, and the most underused, is the state’s power as a purchaser. A government buying care on behalf of a population can set the price it pays, exercising a bargaining power that no individual patient at a hospital admissions desk will ever have. India already does this in pockets: for example, the Central Government Health Scheme sets standardized rates for its listed procedures, and Ayushman Bharat–PMJAY reimburses fixed package rates to empaneled hospitals across the country (4). This is monopsony deployed for public benefit, and it is well grounded in health financing literature, where strategic purchasing and deliberate price-setting are recognized as some of the most effective tools a state has to make care affordable and to steer it toward value (5). The same literature also records the comparative lesson: the middle-income countries that brought out-of-pocket spending down sharply, rather than nibbling at it, did so chiefly by expanding tax-funded public financing, not by waiting for private insurance markets to mature. Thailand’s tax-funded Universal Coverage Scheme, introduced in 2002, is the most cited example. It brought the out-of-pocket share down to a fraction of its former level within a decade; Sri Lanka has long achieved unusually strong financial protection at modest cost through the same public route (5). Each mechanism converts private, unpredictable expenses into collective, governed ones, and the rest of the argument follows from that conversion.

## The budget is still too small, and aimed at the wrong gap

If the direction of travel is right, the scale is not yet. Government health expenditure stand at approximately 1.9% of gross domestic product, short of the 2.5% goal set by the National Health Policy for 2025 (6, 7). This gap matters less as a missed round number than for what it leaves uncovered, and here the conventional picture is misleading. The most catastrophic spending in India does not come from dramatic hospital admissions. It comes from the undramatic, recurring costs of outpatient consultations, medicines, and diagnostics. One national analysis found that catastrophic spending was more common for outpatient care than hospitalization, affecting 47.8% against 43.1% of households, with impoverishment similarly higher for outpatient care (8).

Within that outpatient burden, one component dominates: medicines are the single largest element of out-of-pocket health spending in India, accounting for approximately two-thirds of it according to the most widely cited national estimate (2). This single fact reframes the entire problem. A health system can build hospitals, empanel them and insure admissions, and still leave families impoverished by the chemist’s bill for chronic diseases, because the money that households actually hemorrhage flows through the parts of the system that current financing reaches least. Ayushman Bharat–PMJAY, for all its scale, pays for secondary and tertiary hospitalization, but not for outpatient drugs and tests that drain budgets week after week (4). A larger budget aimed at the wrong target would miss the majority of the burden it is meant to relieve. The headline indicators, gathered in Table 1, tell the same story from several angles at once: protection has improved, but its scale and aim both fall short of the burden.

**Table 1:** Selected health-financing indicators for India.

Indicator	Latest value	Source
Out-of-pocket share of total health expenditure	39.4% (from 64.2% in 2013–14)	(1)
Government share of total health expenditure	48.0% (from 28.6%)	(1)
Government health expenditure (% of GDP)	~1.9% (policy target 2.5%)	(6, 7)
Total health expenditure (% of GDP)	~3.8%	(6)
People pushed into poverty yearly by health costs	~55 million	(2)
Medicines as a share of out-of-pocket spending	approximately two-thirds	(2)

GDP, gross domestic product; THE, total health expenditure.

## What works is already in the country

The encouraging part of this argument is that the remedy need not be invented. India already runs one. Since 1995, the Tamil Nadu Medical Services Corporation has procured essential drugs through a transparent, pooled tender system and distributed them free across the state’s government facilities, with quality controls and stock tracking that keep medicines available at prices a fraction of the branded retail cost (9). The model is widely credited with both lowering out-of-pocket spending and raising the use of public facilities. A peer-reviewed benefit-incidence analysis of national survey data found that public facility utilization rose substantially in Tamil Nadu and Rajasthan, the two free-medicine states, even as it fell in a comparator state that relied on a discounted-sales model instead. The analysis also found that the public subsidy was progressive, reaching the poorest households the most (10). Rajasthan built its scheme on the same logic. Pooled public procurement of this kind is strategic purchasing by another name: the state aggregates demand, sets the price, and removes the charge at the point of care, which is exactly the mechanism that the financing argument predicts should work.

The striking thing is how unevenly this proven idea has spread. Despite the Planning Commission’s High Level Expert Group on Universal Health Coverage recommending a countrywide free essential-medicines and diagnostics program on the pattern of Tamil Nadu and Rajasthan, the majority of states still lack a functioning scheme, and patients continue to buy drugs that the public system could supply far more cheaply (11). The implication for the central argument is precise. The lever that reaches the largest slice of the out-of-pocket burden, free essential medicines delivered through pooled procurement, is not speculative. It has a three-decade track record within India’s own federation. Scaling it is a question of political will and public money, not of evidence, and that is an unusually solvable kind of problem.

## Making the money stick

Money is necessary but not sufficient, and the Tamil Nadu experience is instructive about this difference. What made that model work was not the budget line alone but the machinery around it: an autonomous corporation procuring through open tenders, direct delivery to district warehouses, computerized stock tracking, and quality controls that require suppliers to meet drug standards before payment (9). Pooled procurement without those systems degrades into stockouts and substandard supplies, at which point patients return to private chemists and savings evaporate. The lesson is that a free-medicine guarantee is an operational commitment, not merely a fiscal one.

Two further levers extend the same logic. The first is rational generic substitution: prescribing by generic name and dispensing quality-assured generics, supported by a network of public generic medicine outlets, breaks the link between brand marketing and prices that inflate so much of the medicines bill (11). The second lever is reliable last-mile availability, since a medicine listed as free but absent from the shelf protects no one. The implication for policymakers cuts both ways, demanding and hopeful at once: the binding constraint on reducing the largest component of household health spending is less a shortage of money than the unglamorous work of procurement, supply chains, and quality assurance, all of which India has already built and proven in at least one large state.

## Why families still pay

A serious objection runs deeper than money. Even when a public facility stands nearby and charges nothing, many households walk past it to a private clinic and pay. The private sector still delivers approximately two-thirds of outpatient care in India (12), and the reasons families give are not mysterious: unreliable drug stocks, absent or hurried staff, long waits, inconvenient hours, and plain distrust of the quality on offer. If that is why people pay, then more public money spent in the same way will not lower the household bill; it will simply be bypassed. This is the strongest objection to the argument made here, and it deserves to be met head-on. However, it points not toward private insurance but rather toward what public spending must actually buy. Out-of-pocket costs fall only when the free provision is good enough to be chosen, which is a claim about quality, reliability, and respect as much as about budget lines. It is precisely why Tamil Nadu matters: its free medicines model drew patients back to public facilities and lowered what they spent because the medicines were dependably there (9, 10). The demand side does not refute the financing argument so much as it disciplines it, insisting that the money buy care good enough that families no longer feel they must pay to be treated properly.

## What the extra spending should buy

Where a larger budget should go deserves to be set out plainly, because composition matters more than the headline figure. The first claim on new money should be primary care: a strengthened network of health and wellness centers, properly staffed and stocked, can manage the great majority of everyday illnesses free of charge and close to home, which is the very outpatient burden that drives the majority of catastrophic spending (8). The second is a medicines guarantee, with free essential drugs supplied through pooled procurement on the same pattern as Tamil Nadu, reaching the single largest component of out-of-pocket spending (2, 9). Third is free diagnostics, the quiet companion of medicines in the outpatient bill, since an unaffordable scan or test stops care as surely as an unaffordable drug. None of these are exotic; all three sit within the existing policy vocabulary, and all three are starved of funds rather than of ideas.

A natural objection is that India already has a route to protection in its flagship insurance scheme and that the answer is simply to expand it. However, this misreads the data. Ayushman Bharat–PMJAY insures hospitalization, while the burden that impoverishes families is overwhelmingly from outpatient care (4, 8). Pouring more money into hospitalization coverage, however worthy, would leave the largest leak unplugged and might even widen it if a generously insured hospital sector pulls clinical activity and public funds toward admission and away from the clinic. Insurance has its place, but it cannot substitute for the public provision of primary care and medicines, and that is the deeper reason why the budget, rather than the premium, is the decisive instrument.

## The fiscal-space question

Why has the 2.5% target slipped for so long? Part of the answer is structural. India raises relatively little in tax as a share of its economy, so every social ministry competes for a thin slice, and health has rarely won that contest against more visible priorities. Part is political: the benefits of financial protection are diffuse and slow to appear, while the costs of higher spending are immediate and concentrated, which is an unpromising arithmetic for any finance minister. However, the framing of health spending as a cost to be minimized is itself the error. The 55 million people pushed into poverty by medical bills each year (2) are a fiscal loss and a human one, in foregone productivity, distress borrowing, and later demand on welfare, a loss that never shows up in the health budget but is paid by the wider economy all the same. Interpreted this way, the road to 2.5% is partly self-financing, and the Economic Survey’s own habit of describing health spending as investment rather than expenditure points in the same direction (6). The constraint is real, but it is as much a failure of accounting as of affordability.

## The equity dividend

There is a distributional reason to favor public financing that the aggregate numbers obscure. Out-of-pocket payment is the most regressive way to fund healthcare, because it asks the same rupee of the poor as of the rich and falls hardest on those least able to pay: the chronically ill, the older adults, rural households, and the informal sector poor (2, 3). Every other financing route, whether tax, prepayment, or pooled insurance, is at least mildly progressive by comparison. Thus, the case for raising public spending is not only that it lowers the average bill but also that it redistributes who bears the cost of illness away from those for whom illness is already most ruinous. A bigger, better-aimed public budget is therefore an equity instrument as much as a fiscal one, and that is why the gains of the past decade are worth defending rather than taking for granted.

## Conclusion: the bedside test

India has shown, with its own data, that public financing and household hardship move in opposite directions. The crossover of the past decade was not an accident of the pandemic; it tracked the state paying for, and pricing, more of the nation’s care. The task now is to finish the job and to judge every additional rupee of public spending by one test: does it reduce the bill a family pays at the bedside or at the chemist’s counter? Measured this way, the priorities choose themselves. Raise public health spending toward and beyond 2.5% of gross domestic product. Aim the increase toward primary care, diagnostics, and, above all, free essential medicines delivered through pooled procurement of the kind Tamil Nadu has run for 30 years. Steward the purchasing cleanly and keep the public supply reliable enough that families are not pushed back into paying for what the system promised to provide. None of this is novel in the sense of being untried. It is novel only in that it is being taken seriously, at scale, as the most direct protection India can offer the tens of millions for whom illness still means impoverishment.

## Data Availability

The original contributions presented in the study are included in the article/supplementary material, further inquiries can be directed to the corresponding author.
